# Optimization of Ultrasonic-Microwave Assisted Extraction and Hepatoprotective Activities of Polysaccharides from *Trametes orientalis*

**DOI:** 10.3390/molecules24010147

**Published:** 2019-01-02

**Authors:** Yi Zheng, Jue Cui, An-Hui Chen, Zhi-Min Zong, Xian-Yong Wei

**Affiliations:** 1 Key Laboratory of Coal Processing and Efficient Utilization, Ministry of Education, China University of Mining & Technology, Xuzhou 221116, China; yzuzheng@163.com (Y.Z.); wei_xianyong@163.com (X.-Y.W.); 2 Jiangsu Key Laboratory of Food Resource Development and Quality Safety, Xuzhou University of Technology, Xuzhou 221018, China; cuijue1980@hotmail.com (J.C.); chenah201@163.com (A.-H.C.); 3 State Key Laboratory of High-efficiency Utilization and Green Chemical Engineering, Ningxia University, Yinchuan 750021, China

**Keywords:** *Trametes orientalis*, polysaccharides, ultrasonic-microwave assisted extraction, response surface methodology, liver injury, hepatoprotective activity

## Abstract

Ultrasonic-microwave assisted extraction (UMAE) of *Trametes orientalis* polysaccharides was optimized by response surface methodology. Hepatoprotective effects of a purified *T. orientalis* polysaccharide (TOP-2) were evaluated by alcohol-induced liver injury model mice. The optimal UMAE parameters were indicated as below: ratio of water to raw material 28 mL/g, microwave power 114 W, extraction time 11 min. The polysaccharides yield was 7.52 ± 0.12%, which was well consistent with the predicted value of 7.54%. Pre-treatment with TOP-2 effectively increased the liver index and spleen index in alcohol-treated mice. The elevated serum alanine aminotransferase (ALT) and aspartate aminotransferase (AST) levels of mice after alcohol exposure were inhibited by TOP-2 administration. The liver tumor necrosis factor-α (TNF-α), interleukin-1β (IL-1β) levels have decreased significantly as a result of alcohol exposure, while pre-treatment with TOP-2 could mitigate these consequences. Furthermore, pre-treatment with TOP-2 could efficiently boost the superoxidase dismutase (SOD), catalase (CAT) and glutathione peroxidase (GSH-Px) activities, and observably constrain the malondialdehyde (MDA) level. The findings suggest that TOP-2 might be useful for alleviating the alcohol-induced hepatotoxicity via its antioxidant and anti-inflammatory potential.

## 1. Introduction

Alcoholic liver disease (ALD) is one of the most prevalent liver diseases throughout the world [1]. ALD can progress from fatty liver, simple steatosis to more serious hepatopathy, for instance, hepatitis, fibrosis, cirrhosis, and even hepatocellular carcinoma [2]. There are plenty of probable causes that influence the advancement of ALD, like daily intake, continuation, alcohol category, drinking patterns, sex, nationality, and genetic factors [3]. Presently, there is no satisfactory therapy applicable for patients with ALD [4,5]. In addition, new and safe treatments are urgently necessitated to ameliorate the survival of ALD [6]. It is well known that oxidative stress performs a central character in the advancement of alcohol-induced liver injury [7]. As oxidative stress is important in hepatopathy, exogenous antioxidants are understandably regarded as a promising therapeutic strategy for the treatment of ALD [8]. A great number of antioxidants have drawn considerable attention as promising beneficial supplements to eliminate alcohol-induced liver injury in animal models [9]. Dozens of natural polysaccharides have been described to have substantial hepatoprotective activities against alcohol-induced liver injury [10,11,12,13].

*Trametes orientalis*, a basidiomycetous fungus of the family *Polyporaceae*, has been adopted as a traditional Chinese medicine for the treatment of pulmonary disease [14,15]. We obtained an isolated polysaccharide fraction (TOP-2) from *T. orientalis*. TOP-2 was found to consist of galactose, glucose, mannose, and arabinose in the molar ratios of 5.79:5.77:3.45:1, with an average molecular weight of 63 kDa. TOP-2 exhibited potent antioxidant capacities in four assays of 1,1-diphenyl-2-picrylhydrazyl scavenging capacity, reducing capacity, superoxide scavenging capacity, and ferrous ions chelating capacity [16]. Moreover, TOP-2 effectively reduced the immunosuppression and oxidative stress generated by cyclophosphamide in mice via its immunoregulation and antioxidation [17]. The above results provide a basis for exploring TOP-2 as a possible antioxidant to ameliorate alcohol-induced liver damage. The first objective of our work was to see if TOP-2 possesses any hepatoprotective activity against liver damage due to alcohol exposure in mice.

Previously, we used ultrasound-assisted extraction to extract polysaccharides from *T. orientalis* [16]. However, we found that it was hard to control the ultrasonic temperature in the UAE procedure. Recently, ultrasonic-microwave assisted extraction (UMAE), fully using of high-energy effect of microwave and ultrasonic cavitation, has been effectively applied for extraction of polysaccharides from different materials, such as mushrooms and herbal plants [18,19,20,21]. Response surface methodology (RSM) is an efficient statistical method for the investigation of complex processes. The second objective of the research was to optimize the UMAE parameters of polysaccharides from *T. orientalis* using RSM.

## 2. Results and Discussion

### 2.1. Single Factor Experiment

#### 2.1.1. Influence of Ratio of Water to Raw Material on the Yield of Polysaccharides

**Figure 1:**
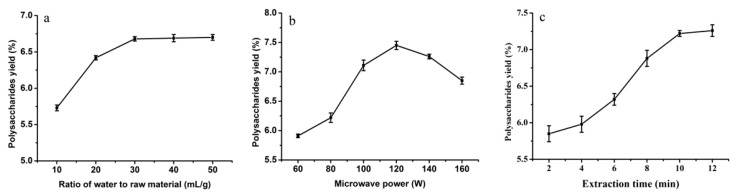
Effects of different extraction parameters ((**a**) ratio of water to raw material, mL/g; (**b**) microwave power, W; and (**c**) extraction time, min) on yield of polysaccharides. (**a**) Microwave power and extraction time were fixed at 100 W and 20 min, respectively. (**b**) Ratio of water to raw material and extraction time were fixed at 20 mL/g and 10 min, respectively. (**c**) Ratio of water to raw material and microwave power were fixed at 20 mL/g and 100 W, respectively. Results are represented as mean ± SD (*n* = 3).

Ratio of water to raw material can significantly influence the yield of polysaccharides. The polysaccharides in the raw material cannot be entirely extracted with an insufficient ratio of water to raw material. Meanwhile, if the ratio of water to raw material is excessive, it will cause an increase in energy consumption. Figure 1a demonstrated the effect of ratio of water to raw material on the yield of *T. orientalis* polysaccharides. The yield of polysaccharides increased with an increase in the ratio of water to raw material under 30 mL/g. However, no palpable variation in the yield was demonstrated as the ratio varied from 30 to 50 mL/g. From our results in Figure 1a, it is suggested that the optimum ratio of water to raw material is around 30 mL/g for UMAE of *T. orientalis* polysaccharides.

#### 2.1.2. Influence of Microwave Power on the Yield of Polysaccharides

Microwave power has a significant effect on the yield of polysaccharides. Figure 1b shows the influence of microwave power on the yield of *T. orientalis* polysaccharides. The yield of polysaccharides rose with increasing microwave power, to a peak at 120 W. Then the yield of polysaccharides declined as the microwave power increased from 120 to 160 W. This may be due to the fact that excess microwave power may result in degradation of polysaccharides. Hence 120 W was initially chosen as the optimal microwave power for UMAE of *T. orientalis* polysaccharides. 

#### 2.1.3. Influence of Extraction Time on the Yield of Polysaccharides

The influence of extraction time on the yield of *T. orientalis* polysaccharides is shown in Figure 1c. The yield of polysaccharides increases significantly with increasing extraction time, reaching a maximum at 10 min. No significant increase in the yield was observed as extraction time increased from 10 to 12 min. From the results in Figure 1c, an optimal extraction time of 10 min was used.

### 2.2. Optimization of Extraction of T. orientalis Polysaccharides

#### 2.2.1. Statistical Analysis and Model Fitting

Based on the results of single factor experiment, the extraction parameters for UMAE of *T. orientalis* polysaccharides were further optimized by a 17-run Box-Behnken design (BBD) with Design Expert 8.0.6 (Stat-Ease Inc., Minneapolis, MN, USA). Table 1 shows the extraction parameters and the yields of *T. orientalis* polysaccharides. 

It was found that a functional relationship of response variable and independent variables could be described as follows:

*Y* = 5.306 − 0.1775 *X*_1_ − 0.165 *X*_2_ + 0.4125 *X*_3_ + 0.43 *X*_1_*X*_2_ + 0.04 *X*_1_*X*_3_ − 0.27 *X*_2_*X*_3_ − 0.718 *X*_1_^2^ − 0.633 *X*_2_^2^ − 0.448 *X*_3_^2^ (1)

where *Y*, *X*_1_, *X*_2_, and *X*_3_ represents the yield of polysaccharides (%), ratio of water to raw material, microwave power, and extraction time, respectively. 

Table 2 gives an analysis of variance (ANOVA) for response surface quadratic model. Model *F*-value of 91.2539, determination coefficient (*R*^2^ = 0.9915) signified that the model was statistically significant. Furthermore, lack of fit was not statistically significant (*p* > 0.05), and Adeq precision that represented the signal to noise ratio was acceptable in view of the ratio greater than 4. The findings reveal that the model could be used to navigate the design space.

The significance of coefficients was described by *p*-value which identified the strength of independent variable. The coefficients of *X*_3_, *X*_1_*X*_2_, *X*_2_*X*_3_, *X*_1_^2^, *X*_2_^2^, and *X*_3_^2^ were highly significant (*p* < 0.001), while those of *X*_1_ and *X*_2_ were statistically significant (*p* < 0.01). Furthermore, the coefficient of *X*_1_*X*_3_ was not statistically significant (*p* > 0.05).

#### 2.2.2. Optimization of Polysaccharides Extraction Parameters

**Figure 2:**
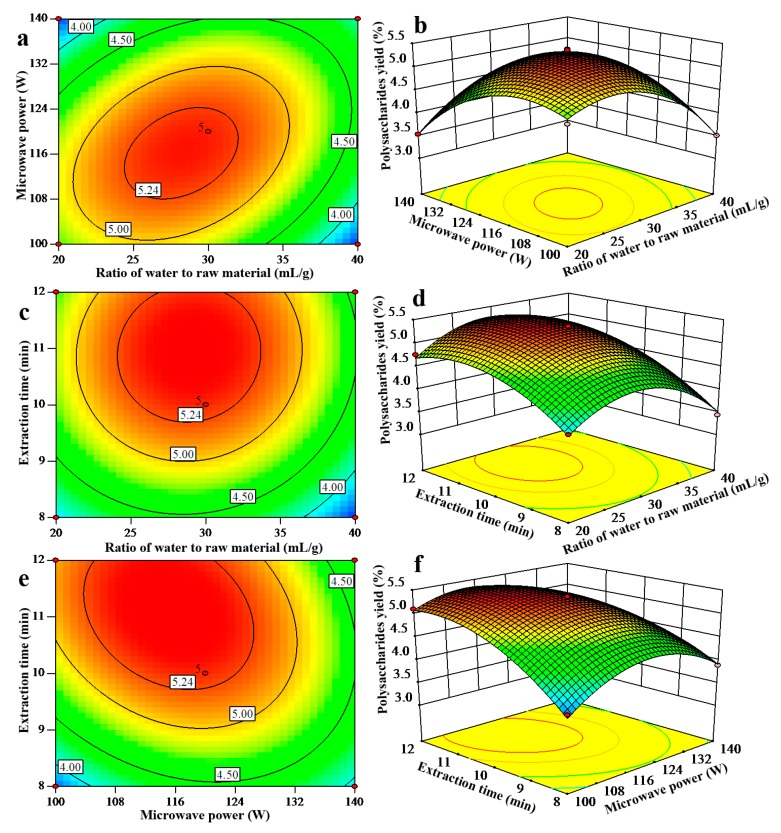
Contour plots and 3D-response surface plots showing the interactive effects of ratio of water to raw material and microwave power (**a**,**b**), ratio of water to raw material and extraction time (**c**,**d**), microwave power and extraction time (**e**,**f**) on yield of polysaccharides.

Figure 2 shows the contour and 3D response surface plots in UMAE of *T. orientalis* polysaccharides. The yield of *T. orientalis* polysaccharides was acquired accompanied with two continuous variables, and the third variable was sustained at zero level. The maximum predicted value was constrained in the smallest ellipse of contour diagram. Elliptical contours will be acquired if there is an attractive interaction between the two independent variables [22].

Figure 2a,b show the interaction between ratio of water to raw material and microwave power on the yield *T. orientalis* polysaccharides, while the extraction time was sustained at zero level. The yield of polysaccharides increased as the ratio of water to raw material increased from 20 to 30 mL/g. Thus, the yield of polysaccharides declined with further increase in the ratio of water to raw material. The yield of polysaccharides also rose with an increase in microwave power ranging from 100 to 114 W, while the yield tended to decrease with increases in microwave power beyond 114 W.

Figure 2c,d display the interaction between ratio of water to raw material and extraction time on the yield *T. orientalis* polysaccharides. The maximum yield of *T. orientalis* polysaccharides was acquired when ratio of water to raw material and extraction time were approximately selected as 30 mL/g and 11 min, respectively. Figure 2e,f exhibit interaction between microwave power and extraction time on the yield *T. orientalis* polysaccharides, while ratio of water to raw material was maintained at zero level. It was found that the yield of *T. orientalis* polysaccharides increased with an increase in microwave power from 100 to 114 W, while the yield of polysaccharides tended to descend with further increase in microwave power. Meanwhile, the yield of polysaccharides rose with an increase in extraction time, reaching a maximum at 11 min. 

#### 2.2.3. Verification of Predictive Model

On account of Equation (1), the optimal extraction parameters for UMAE of *T. orientalis* polysaccharides were indicated as below: ratio of water to raw material 28 mL/g, microwave power 114 W, and extraction time 11 min. The experimental yield of *T. orientalis* polysaccharides was 7.52 ± 0.12% (*n* = 3), showing no difference with the predicted value of 7.54%. These findings indicat that the model of Equation (1) was valid for UMAE of *T. orientalis* polysaccharides.

### 2.3. Comparison with Other Extraction Methods

The yield of *T. orientalis* polysaccharides by UMAE was compared with those by other extraction methods including hot water extraction (HWE), ultrasonic assisted extraction (UAE), and microwave assisted extraction (MAE). The yields of *T. orientalis* polysaccharides by HWE, UAE and MAE were 6.48 ± 0.14%, 7.49 ± 0.11% and 6.25 ± 0.09% where extraction times were 180, 40 and 25 min, respectively. Compared with other extraction methods, UMAE method was characterized by time-saving and high-yield potential which was more valid for extraction of *T. orientalis* polysaccharides.

### 2.4. Hepatoprotective Activities of TOP-2

#### 2.4.1. Influence of TOP-2 on Body Weight and Organ Index

Body weight is regarded as a presumptive barometer of health [23]. As shown in Table 3, the increase of body weight in alcohol-treated mice was significantly lower than that in normal and TOP-2 groups (*p* < 0.05). There was no statistical significance among TOP-2 groups and normal control (*p* > 0.05). 

Hepatomegaly and splenomegaly, as reflected by liver index and spleen index, are frequently pathological findings after the alcohol consumption. Liver index and spleen index in mice exposed to alcohol were significantly higher than those in normal group (*p* < 0.05), while TOP-2 pre-treatment could significantly reduce the liver index and spleen index in comparison with the model mice (*p* < 0.05). Hepatomegaly and splenomegaly were observed in alcohol-treated mice, presumably owing to the accumulation of collagen and fat in the liver and spleen [24]. However, TOP-2 made a remarkable progress in body weight, liver index and spleen index in alcohol-treated mice. Similar ameliorative effects were observed in liver injury mice treated by administration of polysaccharides from *Pleurotus eryngii* SI-04 [25], *Zizyphus jujube cv. Shaanbeitanzao* [26], purple sweet potato [27].

#### 2.4.2. Influence of TOP-2 on Serum Aminotransferase Activities

**Figure 3:**
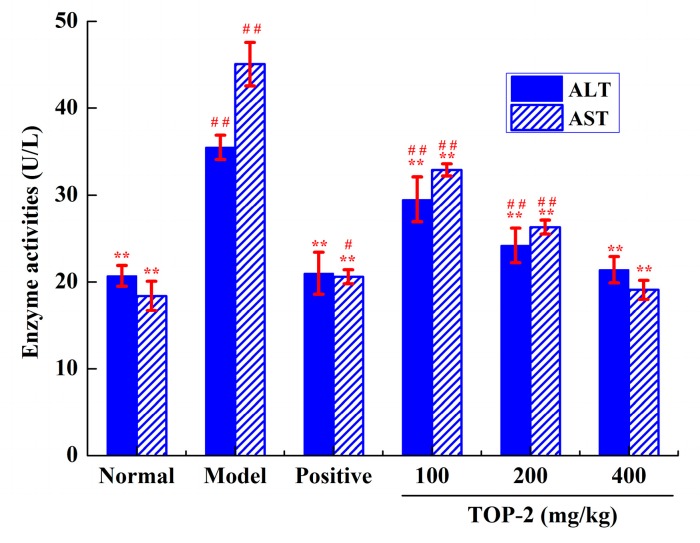
Effects of TOP-2 on serum ALT and AST levels in alcohol-induced liver injury mice. Results are represented as means ± SD (*n* = 10). ** *p* < 0.01, by contrast with model control; ^#^ *p* < 0.05 and ^##^ *p* < 0.01, by contrast with normal control.

In hepatocytes, alcohol-generated ROS are responsible for oxidative stress resulting in hepatocyte injury. Serum alanine aminotransferase (ALT) and aspartate aminotransferase (AST) activities are elevated in ALD; hence serum AST and ALT activities are commonly assessed as biomarkers for alcohol-induced hepatotoxicity [28,29]. As shown in Figure 3, alcohol-treated mice show compelling increase in serum AST and ALT activities in comparison with normal animals (*p* < 0.01), indicating that hepatocyte damage was induced by alcohol and that the alcohol-induced liver damage model had been set up properly. Pre-treatment with TOP-2 for 20 days before alcohol exposure exhibited significant protective effects against alcohol according to significantly decreased serum AST and ALT activities in comparison with model mice (*p* < 0.01), dose-dependently. Moreover, no significant difference existed between the normal mice and the TOP-2 group mice at 400 mg/kg (*p* > 0.05). These findings show that TOP-2 administration could down-regulate the elevated AST and ALT activities back to normal, indicating that TOP-2 had the potential to stabilize the plasma membrane, thereby protecting the structural integrity of hepatocytes from alcohol exposure [30]. These consequences are consistent with the previous reports [31,32].

#### 2.4.3. Influence of TOP-2 on Liver Cytokine Levels

Pro-inflammatory cytokines, for instance, tumor necrosis factor-α (TNF-α) and interleukin-1β (IL-1β), act a pivotal character in the initiation and development of alcoholic liver injury [33]. In liver, TNF-α is primarily generated by activated Kupffer cells and is engaged in the pathophysiology of alcoholic liver injury [34]. IL-1β is another pro-inflammatory cytokine, which enhances pro-inflammatory cytokine secretion, sensitizes hepatocytes to death signals, induces hepatic steatosis, and advances liver fibrosis [35]. 

**Figure 4:**
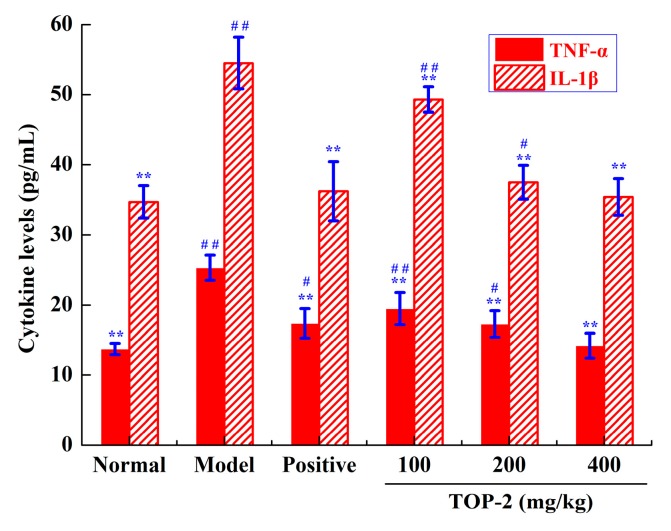
Effects of TOP-2 on liver TNF-α and IL-1β levels in alcohol-induced liver injury mice. Results are represented as means ± SD (*n* = 10). ** *p* < 0.01, by contrast with model control; ^#^ *p* < 0.05 and ^##^ *p* < 0.01, by contrast with normal control.

Enzyme-linked immunosorbent assay (ELISA) kits were engaged to ascertain the actions of TOP-2 on liver TNF-α and IL-1β levels in alcohol-treated mice. As revealed in Figure 4, the secretions of liver TNF-α and IL-1β in model mice were markedly boosted in comparison with normal mice (*p* < 0.01), signified that exposure to alcohol activated inflammatory reaction in hepatocytes. Pre-treatment with TOP-2 administration significantly inhibited the secretions of liver TNF-α and IL-1β (*p* < 0.01), dose-dependently. The liver TNF-α and IL-1β levels in the TOP-2 group mice at 400 mg/kg restored to normal level (*p* > 0.05). These findings imply that TOP-2 have potential anti-inflammatory effects against alcoholic liver injury by down-regulating the expressions of liver TNF-α and IL-1β, which are in agreement with previous reports [36,37]. Pre-treatment with TOP-2 administration resulted in significant protective effects, which might be on account of stabilizing Kupffer cells and suppressing macrophage activation and prostaglandin production [38].

#### 2.4.4. Influence of TOP-2 on Liver Antioxidant Enzyme Activities and Malondialdehyde (MDA) Levels

Enzymatic antioxidant system is crucial for cellular response in the cause of tackling oxidative stress under physiological states. A large number of studies have shown that antioxidant enzymes and the level of lipid peroxidation are noticeably influenced by alcohol intake in various animal models [39]. Effects of TOP-2 on liver superoxidase dismutase (SOD), catalase (CAT), glutathione peroxidase (GSH-Px) activities and MDA levels in alcohol-induced liver injury mice are reveal in Table 4. 

Administration of alcohol significantly reduced the SOD, CAT, and GSH-Px activities in comparison with normal mice (*p* < 0.01). All the enzymes activities in mice pre-treated with TOP-2 were significantly higher than those in model mice (*p* < 0.01). The liver SOD and GSH-Px activities in the TOP-2 group at 400 mg/kg were restored to normal levels (*p* > 0.05). Alcohol-treated mice showed a significant increase in the MDA levels as compared with normal mice (*p* < 0.01). The MDA levels in mice pre-treated with TOP-2 were significantly lower than those in model mice (*p* < 0.01). The MDA levels in the TOP-2 group at 400 mg/kg were restored to normal levels. The results indicate that TOP-2 have the potential to protect mice against alcohol-induced oxidative stress. The hepatoprotective effects of TOP-2 are associated with its antioxidant activities; however, the detailed molecular mechanisms should be further researched.

## 3. Materials and Methods 

### 3.1. Materials and Chemicals

Fruiting bodies of *T. orientalis* were obtained from Jilin Province, China. DE-52 ion-exchange cellulose and Sephadex G-100 were obtained from General Electric Healthcare (Fairfield, CT, USA). ALT, AST, TNF-α, IL-1β, CAT, GSH-Px, SOD and MDA reagent kits were acquired from Nanjing Jiancheng Bio-engineering Institute (Nanjing, China). Silibinin was acquired from Tianjin Tasly Sants Pharmaceutical Co. Ltd. (Tianjin, China). 

### 3.2. UMAE of T. Orientalis Polysaccharides

#### 3.2.1. UMAE Process

The extraction process of *T. orientalis* polysaccharides was conducted in an UMAE equipment (CW-2000, Shanghai Xintuo Analytical Instruments Co. Ltd., Shanghai, China). Ultrasonic power was fixed at 50 W. After the UMAE, the extract solutions were centrifuged (10 min at 3500 r/min) to obtain the supernatants. The supernatants were amalgamated, concentrated using a rotary evaporator (R201L, Shanghai SENCOTechnology Co., Ltd., Shanghai, China), and deposited by the addition of dehydrated ethanol to a final concentration of 80% (*v*/*v*) at 4 °C for 12 h. Then the polysaccharides were acquired by centrifugation at 3500 r/min for 10 min, respectively washed by anhydrous ethanol, acetone and aether, and lyophilized by a freeze-dryer (LGJ-18A, Beijing Four-Ring Science Instrument Plant Co., Ltd., Beijing, China). The polysaccharide content was gauged by phenol-sulfuric acid method [40]. The polysaccharides yield (%) was estimated by a formula as follows:

polysaccharides yield (%) = weight of polysaccharides/weight of sample × 100(2)

#### 3.2.2. Single Factor Experiment

A single factor experiment was employed to preparatorily measure the appropriate ranges of extract parameters including ratio of water to raw material, microwave power, and extraction time for the preparation of *T. orientalis* polysaccharides, as displayed in Table 5.

#### 3.2.3. Box-Behnken Design

A three-level three-variable BBD was used to obtain the optimal extraction parameters, as shown in Table 6. The experimental design was made up of 17 experimental points performed randomly.

Extraction yields (*Y*) were counted as the responses for various experimental designs. A multiple regression method was used to evaluate the BBD experimental data for fitting the quadratic polynomial model indicated as below:

(3) $$Y=\beta_{0}+\sum_{i=1}^{3}\beta_{i}X_{i}+\sum_{i<j}^{2}\sum_{j}^{3}\beta_{ij}X_{i}X_{j}+\sum_{i=1}^{3}\beta_{ii}X_{i}^{2}$$

where *X*_i_ and *X*_j_ are the coded independent variables. *Y* and *β*_0_ are the predicted response and intercept, respectively. *β*_i_, *β*_ij_, and *β*_ii_ are the regression coefficients of the linear term, quadratic term, and interactive term, respectively. 

### 3.3. Comparison with Other Extraction Methods

Control experiments of extraction of *T. orientalis* polysaccharides were conducted by hot water extraction (HWE), ultrasonic assisted extraction (UAE), and microwave assisted extraction (MAE), respectively. HWE of *T. orientalis* polysaccharides was conducted with the following parameters: ratio of water to raw material 30 mL/g, extraction temperature 90 °C, and extraction time 3 h. In reference to previous reports [16], UAE of *T. orientalis* polysaccharides was performed with the following parameters: ratio of water to raw material 30 mL/g, ultrasonic power 110 W, extraction temperature 40 °C, and extraction time 40 min. MAE of *T. orientalis* polysaccharides was run with the following extraction parameters: ratio of water to raw material 30 mL/g, microwave power 200 W, and extraction time 25 min.

### 3.4. Purification of T. orientalis Polysaccharides 

The crude *T. orientalis* polysaccharides were isolated and purified according to the previous method [16,17]. Concisely, the crude polysaccharides (20 g/L) were preliminarily isolated by DE-52 ion-exchange cellulose chromatography (2.6 cm × 30 cm, Xiamei Biochemical Science Techne Development Co. Ltd., Shanghai, China) stepwisely eluted with 0, 0.1, 0.3 and 0.5 mol/L sodium chloride solutions (flow rate: 1 mL/min). Consequently, a predominant polysaccharide fraction eluted with 0.1 mol/L NaCl was collected and further purified by Sephadex G-100 chromatography (2.6 cm × 60 cm, Xiamei Biochemical Science Techne Development Co. Ltd.) eluted with deioned water (flow rate: 0.5 mL/min) to obtain TOP-2.

### 3.5. Hepatoprotective Activities of TOP-2

#### 3.5.1. Animal Treatment

The male Kunming mice (20 ± 2 g) were obtained from Jinan Pengyue Laboratory Animal Breeding Co., Ltd (Jinan, China), and maintained at 22 °C. All experiments were operated in the light of the Guide for Care and Use of Laboratory Animals [41]. After an acclimatization period (7 days), the mice were randomly divided into six groups (*n* = 10), including normal group, model group, positive group (silibinin, 200 mg/kg) and TOP-2 pre-treated groups (100, 200 and 400 mg/kg). Positive and TOP-2 mice were intragastrically administered with silibinin and TOP-2, respectively, lasting 20 days. The other mice were intragastrically administered with normal saline. At the 21st day, all the groups except the normal were intragastrically administered with 12 mL/kg of 50% alcohol every twelve hours for three times.

The mice were weighed and killed by decapitation twelve hours after the last intragastrical administration. Whole bloods were collected and centrifuged (10 min at 3500 r/min) to obtain the serums. Liver and spleen were removed, washed, and weighed instantly. The liver was homogenized in nine volumes (of the liver wet weight) of ice-cold normal saline. The homogenate supernatant was prepared by centrifugation at 3500 r/min for 10 min. Organ index was estimated by a formula indicated as below:

organ index (mg/g) = organ weight /body weight(4)

#### 3.5.2. Biochemical Assays

Activities of serum ALT and AST and levels of liver SOD, CAT, GSH-Px, TNF-α, IL-1β and MDA were estimated by reagent kits in the light of the manufacturer’s specifications.

### 3.6. Statistical Analysis

All results were expressed as mean ± SD. Dunnett-t test was used for determining significant differences where *p* < 0.05 and *p* < 0.01 were accepted to be significant. SPSS 21 (IBM SPSS, Armonk, NY, USA) was utilized for statistical analysis.

## 4. Conclusions

UMAE is a time-saving and high-yield method for extraction of *T. orientalis* polysaccharides. The optimal extraction parameters for UMAE of *T. orientalis* polysaccharides obtained by the single-factor and BBD analyses were listed as below: ratio of water to raw material 28 mL/g, microwave power 114 W, and extraction time 11 min. The experimental yield of *T. orientalis* polysaccharides was 7.52 ± 0.12% (*n* = 3) that was in accordance with the predicted value of 7.54%. 

TOP-2 exhibited hepatoprotective actions against alcohol-induced liver injury in mice, which were ascribed to its anti-inflammatory and antioxidant potential. Pre-treatment with TOP-2 effectively increased the liver index and spleen index in alcohol-treated mice. TOP-2 could down-regulate the elevated AST and ALT activities back to normal, suppress the expressions of liver TNF-α and IL-1β, boost the SOD, CAT, and GSH-Px activities, dwindle the MDA levels. TOP-2 might be regarded as an engaging entrant for alcohol induced liver injury and other diseases brought about by oxidative stress and disproportionate inflammatory response.

## Figures and Tables

**Table 1:** The Box-Behnken design matrix and the results for extraction yield of *T. orientalis* polysaccharides.

Run	*X*_1_ (Ratio of Water to Raw Material, mL/g)	*X*_2_ (Microwave Power, W)	*X*_3_ (Extraction Time, min)	Yield (%)
1	0 (30)	0 (120)	0 (10)	7.25
2	−1 (20)	0 (120)	−1 (8)	5.97
3	1 (40)	0 (120)	−1 (8)	5.44
4	−1 (20)	−1 (100)	0 (10)	6.63
5	0 (30)	0 (120)	0 (10)	7.38
6	1 (40)	0 (120)	1 (12)	6.39
7	1 (40)	1 (140)	0 (10)	6.14
8	0 (30)	0 (120)	0 (10)	7.36
9	0 (30)	−1 (100)	−1 (8)	5.78
10	1 (40)	−1 (100)	0 (10)	5.51
11	0 (30)	0 (120)	0 (10)	7.19
12	0 (30)	1 (140)	−1 (8)	5.89
13	0 (30)	1 (140)	1 (12)	6.13
14	−1 (20)	1 (140)	0 (10)	5.54
15	−1 (20)	0 (120)	1 (12)	6.76
16	0 (30)	0 (120)	0 (10)	7.35
17	0 (30)	−1 (100)	1 (12)	7.10

**Table 2:** Analysis of variance (ANOVA) for response surface quadratic model.

Source	Sum of Squares	df	Mean Square	*F* Value	Prob > *F*	Significance
Model	8.0979	9	0.8998	91.2539	<0.0001	***
*X*_1_	0.2520	1	0.2520	25.5629	0.0015	**
*X*_2_	0.2178	1	0.2178	22.0892	0.0022	**
*X*_3_	1.3612	1	1.3612	138.0578	<0.0001	***
*X*_1_ *X*_2_	0.7396	1	0.7396	75.0101	<0.0001	***
*X*_1_ *X*_3_	0.0064	1	0.0064	0.6491	0.4469	ns
*X*_2_ *X*_3_	0.2916	1	0.2916	29.5740	0.0010	***
*X*_1_^2^	2.1706	1	2.1706	220.1448	<0.0001	***
*X*_2_^2^	1.6871	1	1.6871	171.1067	<0.0001	***
*X*_3_^2^	0.8451	1	0.8451	85.7068	<0.0001	***
Residual	0.0690	7	0.0097			
Lack of fit	0.0421	3	0.0140	2.0852	0.2449	ns
Pure error	0.0269	4	0.0067			
Cor total	8.1667	16				
*R* ^2^	0.9915					
*R* ^2^_Adj_	0.9807					
Adeq precision	23.58					

*** Highly significant, *p* < 0.001; ** Very significant, *p* < 0.01; ns non-significant, *p* > 0.05.

**Table 3:** Effects of TOP-2 on increase of body weight and organ index in alcohol-induced liver injury mice.

Group	Dose (mg/kg)	Increase of Body Weight (g)	Liver Index (mg/g)	Spleen Index (mg/g)
Normal		7.57 ± 0.54 *	42.13 ± 2.25 *	4.22 ± 0.34 *
Model		5.32 ± 0.63	57.46 ± 2.74 ^#^	5.05 ± 0.56 ^#^
Positive	200	7.18 ± 0.72 *	44.68 ± 3.21 *	4.28 ± 0.31 *
TOP-2	100	6.33 ± 0.57 *^,#^	50.85 ± 3.37 *^,#^	4.36 ± 0.47 *
TOP-2	200	7.37 ± 0.68 *	43.29 ± 4.06 *	4.27 ± 0.18 *
TOP-2	400	7.48 ± 0.55 *	42.23 ± 3.40 *	4.31 ± 0.36 *

Results are represented as means ± SD (*n* = 10). * *p* < 0.05, by contrast with model control. ^#^ *p* < 0.05, by contrast with normal control.

**Table 4:** Effects of TOP-2 on liver SOD, CAT, GSH-Px activities and MDA levels in alcohol-induced liver injury mice.

Group	Dose (mg/kg)	SOD (U/mg pro)	CAT (U/mg pro)	GSH-Px (U/mg pro)	MDA (nmol/mg pro)
Normal		289.56 ± 5.8 **	60.13 ± 2.8 **	566.09 ± 34 **	0.73 ± 0.04 **
Model		196.43 ± 11 ^##^	50.72 ± 6.9 ^##^	392.32 ± 28 ^##^	1.26 ± 0.07 ^##^
Positive	200	226.73 ± 23 **^,##^	55.52 ± 1.3 **^,##^	471.37 ± 30 **^,##^	0.83 ± 0.06 **^,##^
TOP-2	100	224.34 ± 15 **^,##^	54.64 ± 1.7 **^,##^	432.21 ± 14 **^,##^	0.97 ± 0.08 **^,##^
TOP-2	200	253.61 ± 12 **^,##^	57.34 ± 3.8 **^,##^	487.32 ± 32 **^,##^	0.83 ± 0.16 **^,##^
TOP-2	400	280.45 ± 12 **	60.22 ± 5.8 **	512.53 ± 24 **^,##^	0.78 ± 0.14 **

Results are represented as means ± SD (*n* = 10). ** *p* < 0.01, by contrast with model control. ^##^ *p* < 0.01, by contrast with normal control.

**Table 5:** Independent variables and their levels used for single factor experiment.

Independent Variables	Levels	Extraction Parameters
Ratio of water to raw material (mL/g)	10, 20, 30, 40, 50	Microwave power 100 W and extraction time 20 min
Microwave power (W)	60, 80, 100, 120, 140, 160	Ratio of water to raw material 20 mL/g and extraction time 10 min
Extraction time (min)	2, 4, 6, 8, 10, 12	Ratio of water to raw material 20 mL/g and microwave power 100 W

**Table 6:** Independent variables and their levels used for Box-Behnken design (BBD).

Independent Variables	Symbol	Levels		
		−1	0	1
Ratio of water to raw material (mL/g)	*X*_1_	20	30	40
Microwave power (W)	*X*_2_	100	120	140
Extraction time (min)	*X*_3_	8	10	12
